# Prognostic Factors Affecting Survival in Patients With Head and Neck Adenoid Cystic Carcinoma: A Single-Institution Experience

**DOI:** 10.7759/cureus.100527

**Published:** 2025-12-31

**Authors:** Jereme Y Gan, Kevin Nguyen, Michael S Elliott, Carsten E Palme, Kerwin Shannon, Jonathan Clark, James Wykes, Ruta Gupta, Hubert T Low

**Affiliations:** 1 Otorhinolaryngology, Tan Tock Seng Hospital, Singapore, SGP; 2 Otolaryngology - Head and Neck Surgery, Peter MacCallum Cancer Centre, Melbourne, AUS; 3 Otolaryngology - Head and Neck Surgery, Chris O’Brien Lifehouse, Camperdown, AUS; 4 General Surgery, Chris O’Brien Lifehouse, Camperdown, AUS; 5 Pathology, Chris O’Brien Lifehouse, Camperdown, AUS

**Keywords:** adenoid cystic carcinoma, bone invasion, lymph node involvement, perineural invasion, radiation, recurrence

## Abstract

Background

Head and neck adenoid cystic carcinoma (AdCC) is an uncommon salivary malignancy characterised by indolent growth but aggressive local behaviour, frequent perineural invasion, and late distant metastasis. The prognostic value of lymph node involvement, perineural invasion, bone invasion, and tumour grade remains uncertain. This study evaluated survival outcomes following surgery for AdCC and assessed the association of key pathological factors with disease control.

Methods

A retrospective review of 89 patients with AdCC treated surgically between 1990 and 2020 was performed. Clinicopathological variables were analysed for their impact on overall survival (OS) and disease-free survival (DFS). Survival probabilities were calculated using the Kaplan-Meier method.

Results

Of the 89 patients, 53 (60%) were female, with a mean age of 54.3 ± 15.9 years. Most tumours originated in major salivary glands. Neck dissection was performed in 29 patients, and lymph node metastasis was identified in eight (28%). Perineural invasion was present in 54 (60%) cases. Thirty-one patients (35%) developed recurrence: nine (29%) local, four (12%) regional, and 18 (58%) distant failures. Regional nodal metastasis and high-grade tumours were significantly associated with poorer OS and DFS.

Conclusion

In this cohort, lymph node involvement and tumour grade independently predicted reduced OS and DFS. One-third of patients experienced recurrence, predominantly at distant sites, reinforcing the need for long-term surveillance and improved systemic strategies.

## Introduction

Adenoid cystic carcinoma (AdCC) is an uncommon malignant neoplasm of glandular tissue characterised by indolent growth yet aggressive local behaviour and a propensity for perineural spread. Although most frequently arising in the head and neck region, AdCC may also occur in the lower respiratory tract, skin, breast, cervix, and prostate.

Epidemiological data from the Surveillance, Epidemiology, and End Results (SEER) registry suggest that the incidence of AdCC has declined in recent decades [1]. European and UK centres report annual incidence rates of approximately one per 100,000 population [2,3]. In the United States, almost 700 new cases of head and neck AdCC are diagnosed annually, accounting for approximately 1% of all head and neck cancers [1,4]. AdCC accounts for up to 31% of malignant tumours arising from the minor salivary glands; however, AdCC itself arises in both major and minor salivary glands, with site distribution varying across cohorts [5]. Australian registry data indicate approximately 330 new cases of salivary gland cancer each year, although AdCC-specific reporting remains limited [6].

AdCC occurs across a wide age range but most commonly affects individuals between 40 and 60 years, with near-equal gender distribution [6]. Its natural history contrasts sharply with squamous cell carcinoma, characterised by relatively prolonged survival despite high rates of late recurrence [7]. Histopathological classification reflects biological behaviour: Grade I tumours exhibit tubular or cribriform architecture without solid components; Grade II tumours contain <30% solid areas; and Grade III tumours display predominantly solid patterns [8,9]. The World Health Organization recently introduced a fourth category, high-grade transformation, to denote a distinct, aggressive variant [10].

Surgical resection remains the primary treatment modality, often followed by adjuvant radiotherapy in the presence of adverse features. Chemotherapy plays a limited role and is generally reserved for advanced, metastatic, or palliative settings or clinical trial contexts [11-13].

Reported survival rates remain favourable, with estimated 5-, 10-, and 15-year overall survival rates of approximately 90%, 80%, and 70%, respectively [1]. However, late recurrence is common. An early Australian series of 30 cases reported a 37% local recurrence rate following surgery [14]. International single-institution cohorts have described 10-year disease-specific survival as high as 93%, yet local or distant recurrence occurs in 25-31% of patients, often many years after treatment [7,15,16]. This underscores the need for long-term surveillance.

This study presents a retrospective review of surgically treated AdCC of the head and neck from the Sydney Head and Neck Cancer Institute database. We aimed to evaluate survival outcomes and identify clinicopathological features associated with adverse prognosis.

## Materials and methods

Study design and patient selection

This was a retrospective cohort study of patients with histologically confirmed AdCC of the head and neck treated surgically between January 1990 and December 2020. Eligible patients were identified from the prospectively maintained Sydney Head and Neck Cancer Institute (SHNCI) database. Only patients who underwent primary surgical resection of their tumour were included. Patients treated non-surgically or referred for palliative management without resection were excluded. No cases meeting the inclusion criteria were omitted. Clinical information was supplemented by review of medical records, operative notes, and pathology reports. Ethics approval was obtained from the institutional review board (X16-0367).

Data collection and definitions

Demographic variables included age at diagnosis and sex. Cancer-related variables included anatomical subsite, tumour size, histological subtype, histological grade, perineural invasion (PNI), bone invasion, surgical margin status, and regional lymph node involvement.

Margin status was defined as (1) clear: ≥5 mm histological margin; (2) close: <5 mm; and (3) positive: tumour present at cut edge.

PNI was defined as microscopic tumour infiltration along major or minor nerve branches and was extracted from pathology reports. Histological grading was categorised according to WHO criteria into tubular/cribriform (Grade I), mixed tumours with <30% solid component (Grade II), and solid-predominant tumours (Grade III).

Treatment-related variables included whether neck dissection was performed (elective vs therapeutic), type of procedure, histopathological nodal yield, and nodal metastases. Receipt of adjuvant radiotherapy was recorded, including intent and indication (margin positivity, PNI, high grade, or advanced stage). Chemotherapy use, when present, was also recorded.

Follow-up duration was measured from the date of surgery to the last documented review or death. Recurrence was categorised as local, regional, or distant based on clinical, radiologic, or histopathologic confirmation.

Outcome measures

The primary outcomes were overall survival (OS) and disease-free survival (DFS). OS was defined as the time from surgery to death from any cause or the last follow-up. DFS was defined as the time from surgery to the first evidence of clinical or radiological recurrence (local, regional, or distant), death, or last follow-up.

Secondary outcomes included (1) recurrence rate, (2) time to recurrence, (3) pattern of first recurrence, and (4) anatomical recurrence site.

Patients without an event were censored at the date of last review.

Statistical analysis

Statistical analyses were conducted using Statistical Product and Service Solutions (SPSS, version 20; IBM SPSS Statistics for Windows, Armonk, NY). Continuous variables were summarised as means or medians, and categorical variables as counts and percentages.

Survival curves were generated using the Kaplan-Meier method, and differences between groups were assessed using the log-rank test. Univariate analyses were performed to evaluate the association of clinicopathological variables with OS and DFS.

Variables demonstrating statistical significance on univariate analysis or recognised clinical relevance (age, margin status, tumour size, PNI) were included in multivariate Cox proportional hazards regression models. Hazard ratios (HRs) and 95% confidence intervals (CIs) were calculated. Model assumptions were checked, including proportionality and collinearity diagnostics. A two-sided p-value <0.05 was considered statistically significant.

Follow-up protocol

Patients were reviewed post-operatively at least every three to four months for the first two years, biannually to five years, and annually thereafter. Surveillance included clinical examination supplemented by cross-sectional imaging (CT, MRI, or PET-CT) when recurrence was suspected or per routine institutional practice. Distant metastasis was confirmed radiologically or histologically, where feasible.

## Results

Eighty-nine patients underwent surgical resection for AdCC of the head and neck, of whom 53 (60%) were female. The mean age at diagnosis was 54.3 years (SD ± 15.9). Median follow-up for the cohort was 5.2 years (range: 1 month-23 years). Most tumours originated in the major salivary glands (n=51, 57%). Demographic, pathological, and treatment characteristics are summarised in Table 1.

**Table 1:** Demographic, subsite, treatment, and pathological details

Variables	Adenoid cystic carcinoma n=89		Standard Deviation
Age	Mean	54.3	(+/-15.9)
Gender		N	%
	Male	36	40%
	Female	53	60%
Primary Subsite			
	Parotid	19	21%
	Submandibular gland	22	25%
	Sublingual	10	11%
	Oral minor salivary gland	15	17%
	Nasal cavity and paranasal sinus	8	9%
	Orbit	3	3%
	Glottis/Trachea	8	9%
	Others	4	4%
Radiotherapy			
	Yes	67	75%
	No	22	25%
Neck Dissection			
	Yes	29	33%
	No	60	67%
Nodal involvement			
	Yes	8	9%
	No	81	91%
Perineural Invasion			
	Yes	54	61%
	No	35	39%
Margin Status			
	Clear	27	30%
	Close	14	16%
	Involved	48	54%
Bone Involvement				
	Yes	16	18%
	No	73	82%

Adjuvant radiotherapy was delivered in 67 patients (75%). Neck dissection was undertaken in 29 patients (33%), with regional lymph node metastasis identified histologically in eight (9%). Perineural invasion was present in 54 (61%) patients, and bone invasion in 16 (18%). Surgical margins were clear in 27 (30%), close in 14 (16%), and involved in 48 (54%). Histologic grade was available for 39 patients, of whom 28 (72%) had Grade I, eight (20%) Grade II, and three (8%) Grade III tumours.

Recurrence

A total of 31 (34%) patients developed recurrence at a median of 4.5 years after surgery (range: 0.2-15.9 years), as shown in Table 2.

**Table 2:** Pattern of recurrence

Recurrence (n=31)	Pattern of recurrence	N	%
	Local	9	29%
	Regional	4	13%
	Distant	18	58%
Site of Distant Recurrence (n = 18)	Site	N	%
	Lung	14	78%
	Liver	2	11%
	Brain	1	6%
	Bone	1	6%

First recurrence was distant in 18 patients (58%), local in nine (29%), and regional in four (13%). The lungs constituted the most common site of distant failure (n=14, 78%), followed by the liver (n=2), the brain (n=1), and the bone (n=1). Among patients with locoregional recurrence, 8/13 (62%) were alive with no evidence of disease following salvage therapy. In contrast, distant relapse carried a poor prognosis, with 12/18 patients (67%) dying of disease.

Survival

Overall survival was 82% at five years and 73% at 10 years. Disease-free survival was 66% at five years and 52% at 10 years. Local control was 88% at both 5 and 10 years; regional control was 96% at five years and 92% at 10 years; distant control was 83% at five years and 72% at 10 years.

Pathological predictors

**Figure 1:**
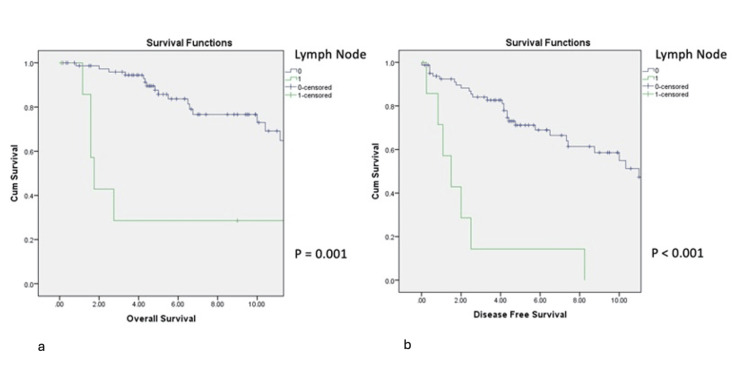
Impact of lymph node involvement on OS (1a) and DFS (1b) DFS: disease-free survival; OS: overall survival

Regional lymph node metastasis at presentation was significantly associated with inferior OS and DFS (p=0.001 and p<0.001, respectively; Figures 1a, 1b).

**Figure 2:**
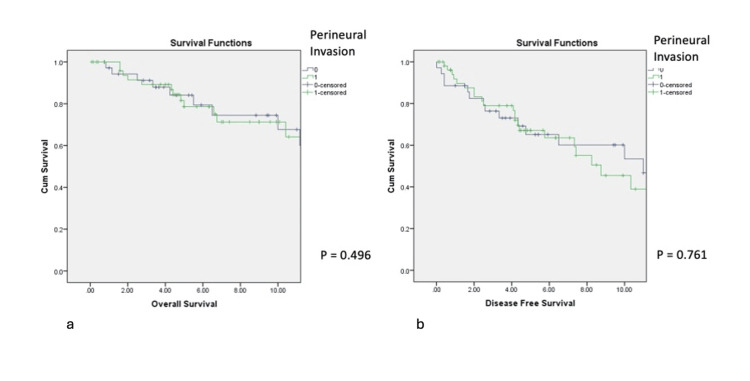
Impact of perineural invasion on OS (2a) and DFS (2b) DFS: disease-free survival; OS: overall survival

Six of the eight patients with nodal disease (75%) subsequently developed distant metastasis, with a median interval to distant failure of 1.2 years (range: 0.8-8.3). Perineural invasion and bone involvement did not significantly affect OS or DFS (Figures 2a, 2b).

**Figure 3:**
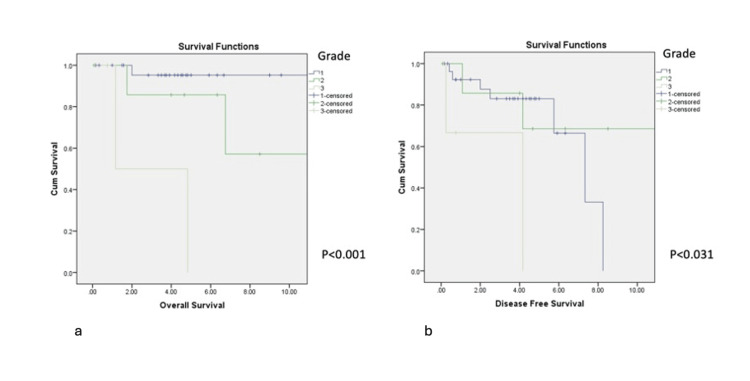
Impact of a grade on OS (3a) and DFS (3b) DFS: disease-free survival; OS: overall survival

The presence of any solid histologic component (Grade II/III) was associated with worse OS (p<0.001) and DFS (p=0.031) compared with Grade I tumours (Figures 3a, 3b). Tumours ≥30 mm in size also demonstrated poorer OS and DFS.

Margin status did not influence survival when adjuvant radiotherapy was administered; patients with involved margins had comparable OS and DFS to those with close or clear margins (p=0.907 and p=0.108, respectively). Tumour subsite (major versus minor salivary gland) was not associated with survival differences (p=0.145 and p=0.670).

On multivariate analysis, increasing age (HR: 1.063, 95% CI: 1.019-1.109, p=0.005), male sex (HR: 2.593, 95% CI: 1.136-5.918, p=0.024), large tumour size (HR: 1.066 per mm, 95% CI: 1.018-1.116, p=0.006), and regional nodal involvement (HR: 5.543, 95% CI: 1.681-18.283, p=0.005) independently predicted poorer OS.

## Discussion

AdCC remains an uncommon malignancy of the head and neck, reflected in the identification of only 89 surgically treated cases over a 30-year period. Consistent with prior reports, nodal metastasis at presentation, larger tumour size (>30 mm), and higher histological grade were associated with poorer outcomes in our cohort. In contrast, PNI, bone involvement, and margin status did not demonstrate a statistically significant influence on survival.

The adverse prognostic impact of nodal involvement in AdCC is well recognised. Zhang et al. reported significantly reduced 5- and 10-year OS among node-positive patients (83.5% vs 57.6% and 77.4% vs 20.6%, respectively), a difference largely attributable to the development of distant metastases [17-19]. In our cohort, approximately three-quarters of node-positive patients subsequently developed distant failure, reinforcing the concept that nodal disease serves as a surrogate marker for systemic dissemination and highlighting the need for improved systemic risk stratification in this subgroup.

Neck management in AdCC remains heterogeneous. In our series, neck dissection was performed selectively, most commonly in patients with clinically evident nodal disease or when vascular access was required for free-flap reconstruction. A systematic review by Luksic et al. reported neck treatment in 42.5% of patients, with occult nodal metastases identified in approximately 14% [20]. Occult nodal disease appears more frequent in oral cavity and oropharyngeal primaries than in major salivary gland tumours, suggesting that elective neck treatment may offer selective benefit in these subsites.

Histological grade emerged as a strong prognostic factor. Tumours containing solid components (Grade II/III) demonstrated significantly worse survival compared with purely tubular or cribriform variants, consistent with multiple institutional series describing the aggressive biological behaviour of solid-predominant disease [21-23]. Tumour size similarly conferred adverse prognosis, in keeping with published evidence identifying disease ≥30 mm as a marker of poorer outcomes [24].

PNI was present in 61% of patients, and close or involved margins were observed in 70%. Despite the high prevalence of these traditionally adverse features, OS remained favourable. This finding likely reflects the widespread use of postoperative radiotherapy in our cohort (75%), which may mitigate the prognostic impact of microscopic residual disease and neural involvement. These results align with prior studies demonstrating variable and sometimes attenuated survival effects of PNI and margin status when modern adjuvant therapy is employed.

Disease recurrence occurred in approximately one-third of patients, most commonly at distant sites, particularly the lung - a hallmark pattern of AdCC. Analysis of National Cancer Database data by Turchan et al. demonstrated that outcomes following distant metastasis vary by site, with lung-only metastases associated with superior survival compared with bone or other visceral involvement [25]. Our cohort size limited the ability to perform similar site-specific survival analyses.

Management of distant recurrence remains challenging. Selected patients with oligometastatic disease may achieve durable disease control with pulmonary metastasectomy or stereotactic ablative radiotherapy [22]. More recently, phase II studies evaluating lenvatinib have reported disease control rates exceeding 85% in recurrent or metastatic AdCC, supporting a role for targeted systemic therapy [8,23]. Advances in the molecular understanding of AdCC - particularly MYB pathway dysregulation - have also prompted early-phase immunotherapeutic strategies, including the MYPHISMO trial evaluating MYB vaccination combined with PD-1 blockade [26,27].

Limitations

This study is limited by its retrospective design, incomplete availability of histological grading in a proportion of cases, and evolving indications for adjuvant radiotherapy over the study period. Nevertheless, the extended duration of follow-up and consistent institutional management strengthen the relevance of these findings and contribute to the ongoing refinement of prognostic stratification in AdCC.

## Conclusions

In this cohort of patients with AdCC, regional lymph node metastasis at diagnosis, larger tumour size, and higher histological grade were independently associated with poorer OS and DFS. In contrast, perineural invasion, margin status, and bone involvement did not demonstrate prognostic significance. Approximately one-third of patients developed recurrence, most commonly at distant sites. These findings reinforce the importance of incorporating nodal status, tumour size, and grade into risk stratification and counselling at presentation and highlight the persistent need for long-term surveillance and systemic control strategies in this disease.
